# Effect of P-Type GaN Buried Layer on the Temperature of AlGaN/GaN HEMTs

**DOI:** 10.3390/mi14071457

**Published:** 2023-07-20

**Authors:** Hanghang Lv, Yanrong Cao, Maodan Ma, Zhiheng Wang, Xinxiang Zhang, Chuan Chen, Linshan Wu, Ling Lv, Xuefeng Zheng, Yongkun Wang, Wenchao Tian, Xiaohua Ma

**Affiliations:** 1 School of Electronics & Mechanical Engineering, Xidian University, Xi’an 710071, China; ymshh@stu.xidian.edu.cn (H.L.); 20041211840@stu.xidian.edu.cn (M.M.); 21041211891@stu.xidian.edu.cn (Z.W.); 22041212821@stu.xidian.edu.cn (X.Z.); 18040100079@stu.xidian.edu.cn (C.C.); 22043222985@stu.xidian.edu.cn (L.W.); ykwang@xidian.edu.cn (Y.W.); wctian@xidian.edu.cn (W.T.); 2 State Key Discipline Laboratory of Wide Bandgap Semiconductor Technology, School of Microelectronics, Xidian University, Xi’an 710071, China; llv@xidian.edu.cn (L.L.); xfzheng@mail.xidian.edu.cn (X.Z.); xhma@xidian.edu.cn (X.M.)

**Keywords:** AlGaN/GaN HEMTs, self-heating effect, channel temperature, channel electric field

## Abstract

In this paper, a P-type GaN buried layer is introduced into the buffer layer of AlGaN/GaN HEMTs, and the effect of the P-type GaN buried layer on the device’s temperature characteristics is studied using Silvaco TCAD software. The results show that, compared to the conventional device structure, the introduction of a P-type GaN buried layer greatly weakens the peak of the channel electric field between the gate and drain of the device. This leads to a more uniform electric field distribution, a substantial reduction in the lattice temperature of the device, and a more uniform temperature distribution. Therefore, the phenomenon of negative resistance caused by self-heating effect is significantly mitigated, while the breakdown performance of the device is also notably enhanced.

## 1. Introduction

Considering the excellent physical properties of gallium nitride (GaN) materials and the excellent properties of two-dimensional electron gas (2DEG) in AlGaN/GaN heterojunctions, gallium nitride high-electron-mobility transistors (HEMTs) have broad prospects in high-temperature, high-voltage, high-frequency, and high-power applications [1,2,3]. Nevertheless, as power density increases, GaN devices generate a large amount of joule heat due to the high peak electric field, resulting in a sharp rise in device temperature and the occurrence of the self-heating effect [4]. The increase in average temperature within GaN transistors and the uneven distribution of dissipated power lead to the formation of hot spots in the submicron region of the channel, which have a serious impact on the output current, gate leakage current, and overall device reliability [5]. The flip chip structure, which can enhance the heat dissipation performance of devices, was proposed by Jiang et al. [6]. A GaN–diamond composite substrate was used to effectively reduce device temperature by Wang et al. [7]. However, the application of GaN devices is still limited by the hot spots due to the nonuniform dissipation of high-power density and the relatively high thermal resistance of the substrate [8,9,10,11]. Furthermore, Miao et al. achieved a reduction of 20 °C in the maximum temperature of the device by inserting a 100 nm graphene layer between the AlGaN barrier layer and GaN buffer layer [12]. Zhou et al. reduced the peak temperature of the device by 15% by growing polycrystalline diamond on the AlGaN/GaN HEMT structure [13]. However, these measures may lead to an increase in the device volume, which is not favorable for the miniaturization and high-density integration of GaN devices. In some studies [14,15], a P-type GaN buried layer (PBL) was used to boost the electrical performance of AlGaN/GaN HEMTs due to the fact that PBL can change the electric field of devices. However, there are currently no research reports on reducing device temperature through PBL modulation of the electric field.

In this paper, using RESURF technology [16], a PBL is introduced into the buffer layer of AlGaN/GaN HEMTs to modulate the electric field between the gate and drain, and Silvaco TCAD software (Atlas 5.34.0.R) is used to study the effect of PBL on the device temperature. The PBL is combined with the N-type GaN buffer to create a structure that can be approximated as a PN junction. This structure partially depletes the 2DEG in the channel, resulting in a reduction in the electron gas density in the corresponding region and an increase in the width of the depletion region of the device. The wider depletion region significantly reduces the maximum electric field at the gate edge near the drain, leading to a more uniform distribution of the electric field between the gate and drain.

## 2. Device Structure and Simulation Setup

**Figure 1:**
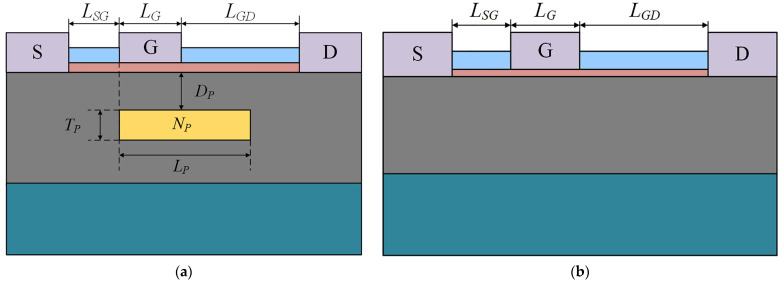
(**a**) Schematic structure of the PBL GaN HEMT. (**b**) Schematic structure of the Cov GaN HEMT.

The AlGaN/GaN HEMT with a PBL (PBL GaN HEMT) is shown in Figure 1a. According to [17] and the actual manufacturing process, the device structure from bottom to top consists of a sapphire substrate, a 1.5 μm GaN buffer layer, a 25 nm AlGaN barrier layer with 30% aluminum composition, and a 150 nm Si_3_N_4_ passivation layer. The distance (*L_SG_*) between the gate and source of the device is 1 μm, the gate length (*L_G_*) is 1 μm, and the distance (*L_GD_*) between the gate and drain is 4 μm. The N-type doping concentration of the GaN buffer layer is 1 × 10^16^ cm^−3^ [18], which is used to simulate the N-type carriers that are inevitably introduced during the fabrication of actual devices. The work function of the gate metal is set to 5.15 eV to form a Schottky contact between the gate and the semiconductor [15]. The conventional AlGaN/GaN HEMT (Cov GaN HEMT) is shown in Figure 1b. This structure is used for comparison with the PBL GaN HEMT, and all structural parameters, except for the PBL, are identical to the PBL GaN HEMT.

The details of key structural parameters of the PBL GaN HEMT and Cov GaN HEMT are summarized in Table 1. As shown in Figure 1a, the left side of the PBL is aligned with the left side of the gate. *L_P_* is the length of the PBL, *T_P_* is the thickness of the PBL, and the *D_P_* represents the spacing between the PBL and the bottom of the AlGaN barrier layer. Furthermore, when using Mg as a P-type dopant, the activation rate is very low due to Mg being a deep-level impurity. Therefore, the hole concentration in the P-type GaN buried layer is unlikely to exceed 1 × 10^18^ cm^−3^ [19] Considering that the simulation should be consistent with reality, *N_P_* is the doping concentration of PBL, and the value is set between 1 × 10^17^ cm^−3^ and 5 × 10^17^ cm^−3^ [20].

**Figure 2:**
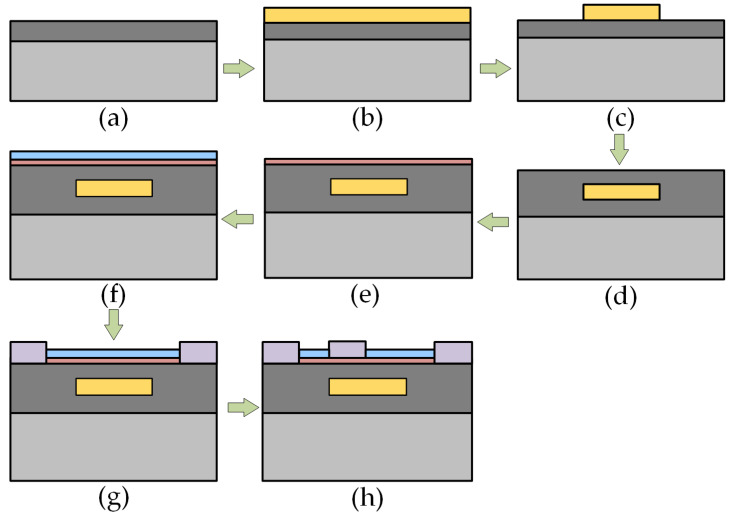
Schematic diagram of the manufacturing process for PBL GaN HEMT.

In order to illustrate how the PBL GaN HEMT can be implemented, Figure 2 presents a schematic diagram of the actual manufacturing process steps for the device.

The corresponding descriptions of the device manufacturing process steps are summarized as follows:

(a) A portion of GaN buffer layer is grown on sapphire substrate via metal–organic chemical vapor deposition (MOCVD).
(b) The P-type GaN buried layer is grown via MOCVD using Mg as a P-type dopant [21].
(c) Using the inductively coupled plasma (ICP) process, the portions of the P-type GaN buried layer on both ends are etched [21].
(d) The remaining GaN buffer layer is grown via MOCVD.
(e) The AlGaN barrier layer is grown on GaN buffer layer via MOCVD.
(f) Using plasma enhanced chemical vapor deposition (PECVD), a Si_3_N_4_ layer is deposited on the AlGaN barrier layer as the passivation layer [20].
(g) The source and drain contact are etched, and then electron beam evaporation is used to deposit Ti/Al/Ni/Au to form the source and drain electrodes, followed by high-temperature annealing.
(h) The gate contact is etched, and then electron beam evaporation is used to deposit Ni/Au, forming the gate electrode.

In the simulation of the GaN device using Silvaco TCAD, the Shockley–Read–Hall (SRH) recombination model was used to simulate the recombination and generation of carriers [22]. The mobility model (Fldmob) accurately describes the variation of carrier mobility under both low and high electric fields, considering the impact of temperature on mobility. The lattice heat flow equation (lat.temp) was used to simulate the transfer of heat within the device, and the thermal conductivity model was used to specify the thermal conductivity of the material in each area of the device. The heat generation model (heat.full) was used to simulate the heat generation in the device during operation, according to the heat flow equation of the following form [23]:

(1) $$H=\left(\frac{\vec{{\left|J_{n}\right|}^{2}}}{q\mu_{n}n}+\frac{\vec{{\left|J_{p}\right|}^{2}}}{q\mu_{p}p}\right)+q\left(R-G\right)\left[\phi_{p}-\phi_{n}+T_{L}\left(P_{p}-P_{n}\right)\right]-T_{L}\left({\vec{J}}_{n}\nabla p_{n}+{\vec{J}}_{p}\nabla P_{p}\right),$$

where *H* is the heat generation, *T_L_* is the local lattice temperature, ${\vec{J}}_{n}$ is the electron current density, ${\vec{J}}_{p}$ is the hole current density, *μ_n_* and *μ_p_* are the mobility of electrons and holes, respectively, *n* and *p* are the concentrations of electrons and holes, respectively, *R* and *G* are the recombination rate and generation rate of carriers, *P_n_* is the absolute thermoelectric power for electrons, and *P_p_* is the absolute thermoelectric power for holes. The right-hand side of Equation (1) consists of three terms: the first term is the joule heating, the second term is the recombination and generation heating and cooling, and the third term denotes the Peltier and Joule–Thomson effects. Compared to joule heating, the effects of the other two terms on heat generation can be neglected. Therefore, Equation (1) can be simplified as follows [24]:

(2) $$H=({\vec{J}}_{n}+{\vec{J}}_{p})\cdot \vec{E},$$

where $\vec{E}$ is the electric field intensity. From Equation (2), it can be observed that the internal heat generation in the device is primarily related to the electric field. Therefore, in the subsequent study on device temperature, the main emphasis is placed on analyzing the changes in the internal electric field of the device.

In addition, thermal conductivity is a physical quantity that measures the ability of a material to conduct heat. Therefore, the thermal conductivity parameters of device materials are important factors that influence heat transfer in the device. In general, the thermal conductivity of a material is temperature-dependent, and the thermal conductivity of different regions of the device during this simulation can be determined using the following model equations [23]:

(3) k(T)=TC.CONST,

(4) $$k\left(T\right)=\left(TC.CONST\right)/{\left(T_{L}/300\right)}^{TC.NPOW},$$

(5) $$k\left(T\right)=1/\left(TC.A+\left(TC.B\right)*T_{L}+\left(TC.C\right)*T_{L}{}^{2}\right),$$

where *k*(*T*) represents the thermal conductivity of the material, *T_L_* is the local lattice temperature, and *TC.CONST*, *TC.NPOW*, *TC.A*, *TC.B*, and *TC.C* are custom model parameters that need to be determined on the basis of the structure and material characteristics of the simulated device. Referring to [25], the model parameters for the thermal conductivity of the device materials are provided in Table 2. The substrate temperature was set to 300 K, and adiabatic conditions were applied to both sides of the device during this simulation.

**Figure 3:**
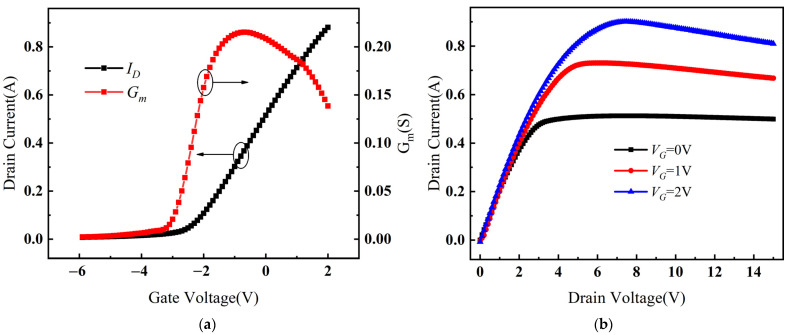
(**a**) The transfer characteristic curves of Cov GaN HEMT. (**b**) The output characteristic curves of Cov GaN HEMT.

The electrical characteristics of Cov GaN HEMTs simulated using these models are shown in Figure 3. The threshold voltage of the device was −2.21 V, the maximum transconductance was 224 mS, and the saturation drain current was 713 mA at *V_G_* = 1 V. These values were compared with the actual device’s −2.18 V, 232 mS, and 728 mA at *V_G_* = 1 V, and the electrical parameters were found to be essentially consistent. Therefore, it can be concluded that the simulation model agreed with the actual device.

## 3. Simulation Results and Discussion

### 3.1. Optimization Design of PBL Structure Parameters

The temperature simulation of PBL GaN HEMT was performed to analyze the effects of PBL structure parameters *L_P_*, *D_P_*, *N_P_*, and *T_P_* on the peak temperature of the channel. Firstly, the initial values of *D_P_* and *N_P_* were set to 0.2 μm and 3 × 10^17^ cm^−3^, respectively. The length *L_P_* and thickness *T_P_* of PBL were then optimized through simulation.

**Figure 4:**
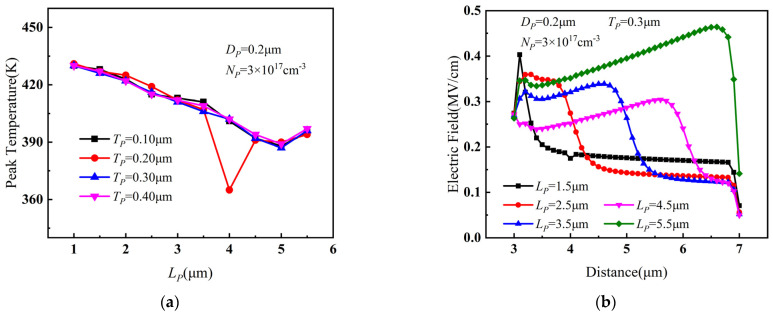
(**a**) Variation of peak temperature with *L_P_* and *T_P_* when *D_P_* = 0.2 μm, *N_P_* = 3 × 10^17^ cm^−3^. (**b**) Variation of channel electric field between gate and drain with *L_P_* when *D_P_* = 0.2 μm, *N_P_* = 3 × 10^17^ cm^−3^, and *T_P_* = 0.3 μm.

The relationship of the peak temperature of PBL GaN HEMTs with *L_P_* and *T_P_* is presented in Figure 4a. It can be observed that, as *L_P_* gradually increased, the peak temperature decreased significantly. However, when *L_P_* continued to increase further, the peak temperature no longer decreased but slightly increased. The variation of channel electric field between gate and drain with *L_P_* is shown in Figure 4b. With the increase in *L_P_*, the depletion effect of PBL on 2DEG was enhanced, leading to a weakening of the channel electric field between the gate and drain, and a reduction in the maximum temperature. However, when *L_P_* became larger, the distance between PBL and the drain decreased, resulting in a sharp increase in the electric field between the right side of PBL and the drain. Therefore, the peak temperature increased marginally.

**Figure 5:**
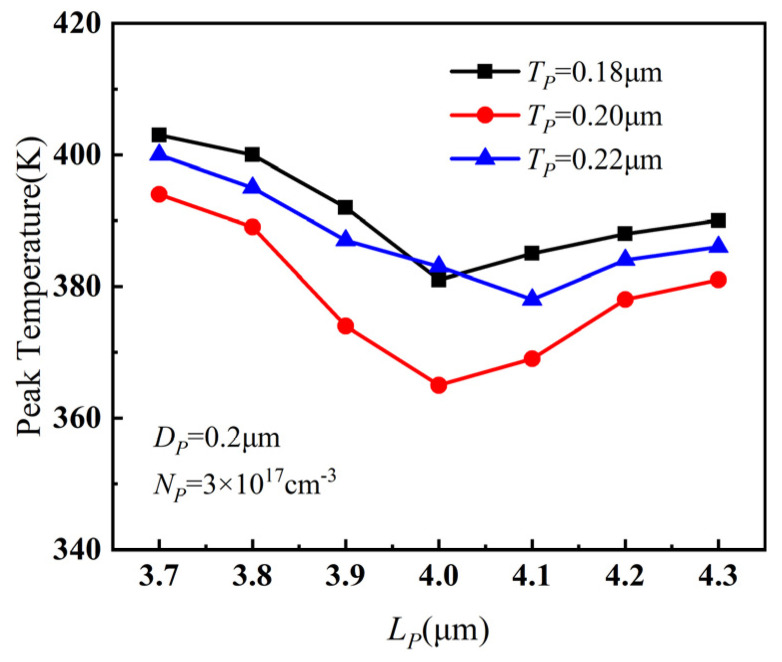
The variation of peak temperature when *T_P_* ranged from 0.18 μm to 0.22 μm and *L_P_* ranged from 3.7 μm to 4.3 μm.

From Figure 4a, it can also be seen that the peak temperature showed only minor variations with different values of *T_P_*. However, when *T_P_* = 0.2 μm and *L_P_* = 4 μm, there was a noteworthy decrease in the device temperature, indicating that the change in *T_P_* had a noticeable impact on the device temperature only when *L_P_* took a specific value. Therefore, a simulation was performed with smaller incremental steps in parameter values near *T_P_* = 0.2 μm and *L_P_* = 4 μm, and the results are shown in Figure 5. As in Figure 4a, the results in Figure 5 also prove that the device temperature was lowest when *T_P_* = 0.2 μm and *L_P_* = 4 μm. Considering the combined effects of *L_P_* and *T_P_* on the device temperature, the optimal value for the length *L_P_* of PBL was determined to be 4 μm, and the optimal value for the thickness *T_P_* was determined to be 0.2 μm.

**Figure 6:**
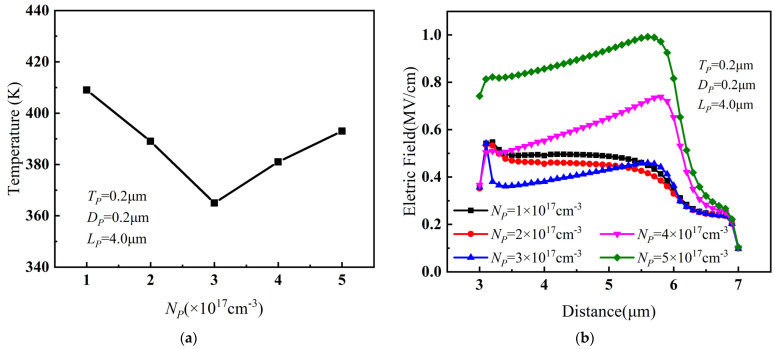
(**a**) The relationship between the peak temperature and *N_P_* when *T_P_* = 0.2 μm, *D_P_* = 0.2 μm, and *L_P_* = 4 μm. (**b**) The relationship between the channel electric field between gate and drain and *N_P_* when *T_P_* = 0.2 μm, *D_P_* = 0.2 μm, and *L_P_* = 4 μm.

Figure 6a shows the relationship between the peak temperature of PBL GaN HEMT and the doping concentration *N_P_* of PBL when *T_P_* = 0.2 μm, *D_P_* = 0.2 μm, and *L_P_* = 4 μm. As *N_P_* increased, the peak temperature decreased greatly. However, after *N_P_* exceeded 3 × 10^17^ cm^−3^, the peak temperature no longer continued to decrease but rather increased noticeably. The relationship of the channel electric field between the gate and drain with *N_P_* is shown in Figure 6b. The electric field initially decreased and then increased with the increase in *N_P_*. Furthermore, when *N_P_* exceeded 3 × 10^17^ cm^−3^, the electric field sharply increased, resulting in an obvious increase in the peak temperature instead. Considering the variations of device temperature and channel electric field between gate and drain with respect to *N_P_*, the optimal doping concentration of PBL was chosen to be 3 × 10^17^ cm^−3^.

**Figure 7:**
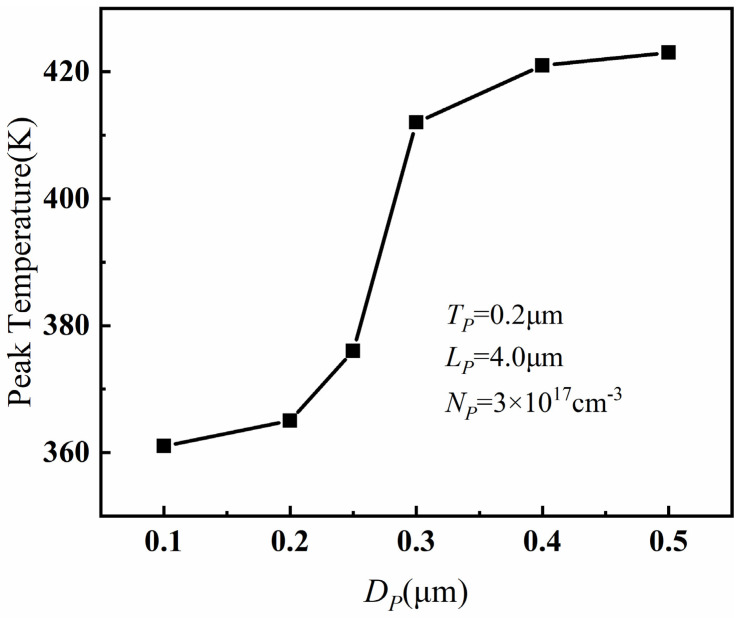
The relationship between the peak temperature and *D_P_* when *T_P_* = 0.2 μm, *L_P_* = 4 μm, and *N_P_* = 3 × 10^17^ cm^−3^.

Figure 7 shows the relationship between the distance *D_P_* between PBL and AlGaN barrier layer and the peak temperature of PBL GaN HEMT when *T_P_* = 0.2 μm, *L_P_* = 4 μm and *N_P_* = 3 × 10^17^ cm^−3^. It can be observed that, with the increase in *D_P_*, the peak temperature increased. This is because, with a larger *D_P_*, the PBL was farther away from the channel, resulting in weaker depletion effect on the carriers. As a result, the electric field modulation capability decreased, and the self-heating effect became stronger, leading to an increase in the peak temperature. Furthermore, there was a violent change in the peak temperature when *D_P_* exceeded 0.25 μm. Analysis of the gate–drain electric field at different *D_P_* values revealed a sharp increase in the electric field between the gate and drain after *D_P_* exceeded 0.25 μm. This ultimately led to a significant variation in the peak temperature, indicating that the influence of PBL on device temperature was most significant when the distance between PBL and the barrier layer was within 0.25 μm. However, *D_P_* should not be too small either, as having PBL too close to the channel can have a significant impact on the device’s electrical performance. Therefore, considering these factors, the optimal value for *D_P_* is 0.2 μm.

### 3.2. The Temperature Performance of PBL GaN HEMT

On the basis of the previous simulation results, the optimal structural parameters for PBL were set as *L_P_* = 4 μm, *D_P_* = 0.2 μm, *N_P_* = 3 × 10^17^ cm^−3^, and *T_P_* = 0.2 μm. Then, temperature simulations were performed for both PBL GaN HEMT and Cov GaN HEMT.

**Figure 8:**
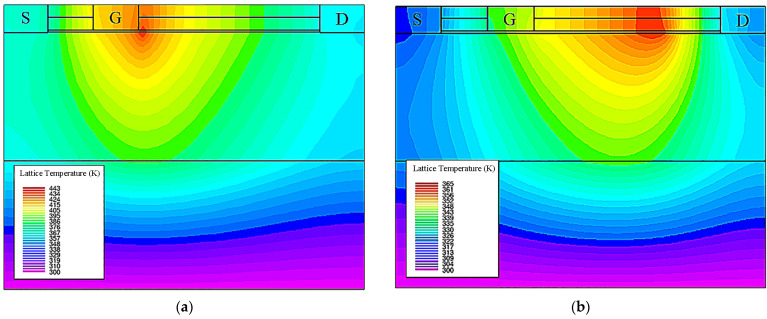
(**a**) The lattice temperature distribution of Cov GaN HEMT. (**b**) The lattice temperature distribution of PBL GaN HEMT.

Figure 8 shows the lattice temperature distribution of Cov GaN HEMT and PBL GaN HEMT devices under the same conditions. The lattice temperature of the PBL GaN HEMT was obviously lower than that of the Cov GaN HEMT, and the temperature distribution was also more uniform. Compared to the concentrated thermal distribution mainly at the gate edge in the Cov GaN HEMT, the thermal distribution in the PBL GaN HEMT was more evenly distributed in the gate–drain region near the drain side.

**Figure 9:**
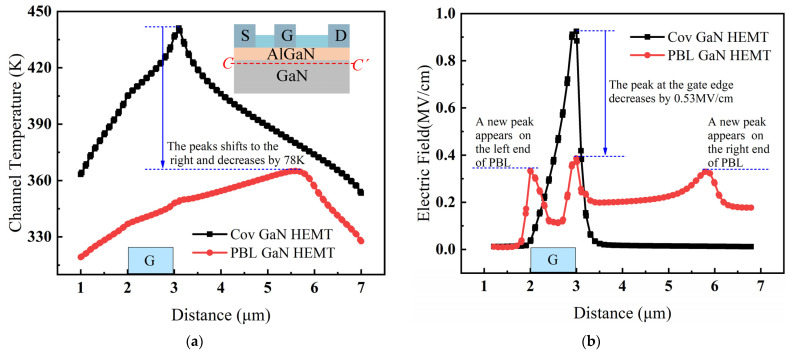
(**a**) The channel temperature extracted along the horizontal line (C–C’) 2 nm below the barrier layer. (**b**) The channel electric field for Cov GaN HEMT and PBL GaN HEMT. Inset: cut line along which channel temperature was extracted.

Figure 9a shows the channel temperature of Cov GaN HEMT and PBL GaN HEMT extracted from the device lattice along the horizontal line C–C’ (2 nm below the barrier layer). It can be clearly seen that the channel temperature distribution of PBL GaN HEMT was more uniform, and the temperature peak shifted greatly to the right. The maximum channel temperature of PBL GaN HEMT was 365 K, while that for Cov GaN HEMT was 443 K. Therefore, the introduction of PBL resulted in a decrease of 78 K in the peak channel temperature of the device. To analyze the decrease in lattice temperature and the change in distribution pattern, the channel electric field was extracted from both devices under the same operating bias, as shown in Figure 9b. It can be observed that the introduction of PBL noticeably reduced the peak electric field at the gate edge, with the maximum electric field decreasing from 0.92 MV/cm to 0.38 MV/cm, resulting in a reduction of 57.6%. Additionally, due to the charge accumulation and shielding effect within GaN devices, two new small electric field peaks were generated at the end of the PBL, which were much lower than the maximum electric field in the Cov GaN HEMT device. Therefore, compared to the Cov GaN HEMT, the PBL GaN HEMT exhibited a noteworthy decrease in the electric field peak, and the presence of these three small electric field peaks made the distribution of the channel electric field more uniform.

**Figure 10:**
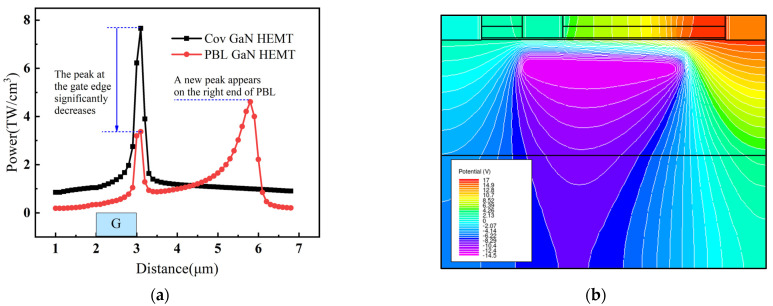
(**a**) The heat power in the channel of Cov GaN HEMT and PBL GaN HEMT. (**b**) The equipotential lines of PBL GaN HEMT.

The heat power in the channel of Cov GaN HEMT and PBL GaN HEMT is shown in Figure 10a. It can be observed that the variation of the channel electric field in PBL GaN HEMT led to a substantial change in the distribution of heat power. The peak heat power at the gate edge decreased, and a new peak was generated at the right end of the PBL, resulting in a more uniform distribution. Therefore, the channel temperature of PBL GaN HEMT in Figure 9a was lower than that of Cov GaN HEMT and exhibited a more uniform distribution. The new peak of heat power at the right end of PBL in Figure 10a was higher than the peak at the gate edge, indicating that the electric field peak at the right end of PBL in Figure 9b played a dominant role in the self-heating effect, resulting in a significant rightward shift of the channel temperature peak in Figure 9a for PBL GaN HEMT. Furthermore, although it can be seen from Figure 9 that the peak electric field magnitude at the gate edge was very close to the peak electric field at the right end of the PBL, it is evident from the equipotential lines in Figure 10b that the equipotential lines near the right end of the PBL were much denser than those at the gate terminal. This indicates that the electric field near the right end of the PBL was larger and more concentrated, resulting in a higher heat power compared to the gate edge.

### 3.3. The Electrical Performance of PBL GaN HEMT

Same as the temperature simulation above, the optimal structural parameters for PBL were set as *L_P_* = 4 μm, *D_P_* = 0.2 μm, *N_P_* = 3 × 10^17^ cm^−3^, and *T_P_* = 0.2 μm. The electrical performance simulations were performed for both PBL GaN HEMT and Cov GaN HEMT.

**Figure 11:**
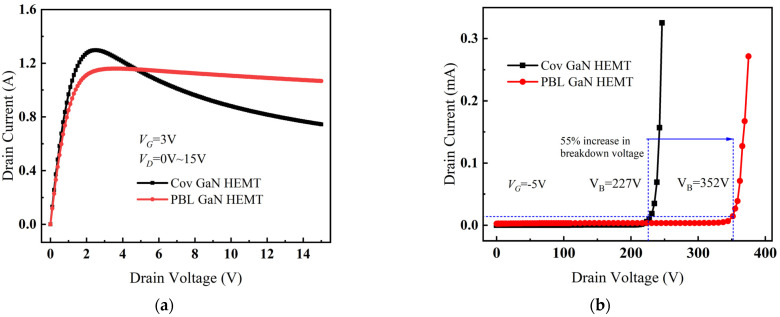
(**a**) The output characteristic curves for Cov GaN HEMT and PBL GaN HEMT. (**b**) The breakdown characteristic curves for Cov GaN HEMT and PBL GaN HEMT.

Figure 11a shows the output characteristics curves of Cov GaN HEMT and PBL GaN HEMT. Due to the partial depletion effect of PBL on the carriers, leading to a decrease in 2DEG density, the saturation drain current of PBL GaN HEMT was slightly reduced, and the on-resistance of PBL GaN HEMT was slightly increased compared to Cov GaN HEMT. Under high-voltage and high-current conditions, the drain current of Cov GaN HEMT decreased significantly after reaching saturation, indicating the occurrence of severe negative resistance due to self-heating [26]. In contrast, the drain current of PBL GaN HEMT only exhibited a slight decrease after saturation, indicating that the introduction of PBL significantly reduced the device temperature and greatly improved the negative resistance phenomenon caused by self-heating.

The breakdown characteristic curves for Cov GaN HEMT and PBL GaN HEMT are shown in Figure 11b, where the breakdown voltage was defined as the drain voltage corresponding to a drain current (*I_D_*) of 0.01 mA when the conductive channel was closed [27]. It can be observed that the breakdown voltage for Cov GaN HEMT was 227 V, while the breakdown voltage for PBL GaN HEMT was 352 V, which represents a 55% improvement. Therefore, the introduction of PBL for electric field modulation not only reduced the device temperature but also enhanced the breakdown performance of the device.

## 4. Conclusions

This paper presented a simulation study on PBL GaN HEMT with a P-type GaN buried layer. The P-type GaN buried layer could modulate the channel electric field distribution between the gate and drain, thereby reducing joule heating and lowering the device temperature. The influence of structural parameters of the P-type GaN buried layer on the device temperature was studied using Silvaco TCAD, and the results showed that when *L_P_* = 4 μm, *D_P_* = 0.2 μm, *N_P_* = 3 × 10^17^ cm^−3^, and *T_P_* = 0.2 μm, the maximum temperature of the device channel decreased from 443 K to 365 K. The negative resistance phenomenon caused by self-heating at high temperatures was significantly improved, and the breakdown characteristics of the device were also noteworthily enhanced.

## Figures and Tables

**Table 1:** The key structural parameters for device simulation.

Parameters	Values
Gate length (*L_G_*)	1 μm
Gate–source spacing (*L_SG_*)	1 μm
Gate–drain spacing (*L_GD_*)	4 μm
Length of PBL (*L_P_*)	1 to 5.5 μm
Thickness of PBL (*T_P_*)	0.1 to 0.4 μm
Doping concentration of PBL (*N_P_*)	1 × 10^17^ to 5 × 10^17^ cm^−3^
Distance between PBL and barrier layer (*D_P_*)	0.1 to 0.5 μm

**Table 2:** The model parameters for the thermal conductivity.

Parameters	Values
*TC.A* (sapphire)	−12.56
*TC.B* (sapphire)	6.81 × 10^−2^
*TC.C* (sapphire)	−7.76 × 10^−5^
*TC.NPOW* (GaN)	1.4
*TC.CONST* (GaN)	1.6
*TC.CONST* (Si_3_N_4_)	0.35
*TC.CONST* (AlGaN)	0.19

## Data Availability

The data presented in this study are available on request from the corresponding author.
